# Mild Oculocutaneous Albinism Type 1B With Subtle Cutaneous Findings: A Dermatology-Oriented Case Report

**DOI:** 10.7759/cureus.106375

**Published:** 2026-04-03

**Authors:** Catalina Montané, Diego Guarda, Priscilla Marquez, Gerardo Bascuñan

**Affiliations:** 1 Dermatology, Universidad de Santiago de Chile, Santiago, CHL; 2 General Medicine, Clínica Andes Salud, Puerto Montt, CHL; 3 General Medicine, Universidad Andres Bello, Santiago, CHL; 4 General Practice, Hospital Familiar y Comunitario de Carahue, Carahue, CHL

**Keywords:** adolescent, oculocutaneous albinism type 1b, photoprotection, preventive dermatology, skin cancer, tyr gene

## Abstract

Oculocutaneous albinism type 1B (OCA1B) may present with subtle dermatologic manifestations and be initially suspected through ophthalmologic abnormalities. We report a 16-year-old female with normal psychomotor development who presented with progressive decreased visual acuity beginning in childhood. Ophthalmologic evaluation demonstrated reduced best-corrected visual acuity (BCVA) and fundoscopic findings, including pale optic discs and increased visibility of choroidal vessels. Suspicion of albinism increased after optical coherence tomography (OCT) revealed the absence of foveal depression. The patient was referred to clinical genetics, where next-generation sequencing (NGS) confirmed *TYR* variants consistent with OCA1B, followed by genetic counseling regarding autosomal recessive inheritance. Dermatologic evaluation later revealed Fitzpatrick phototype II skin and gray-green irides consistent with a mild hypopigmented phenotype of OCA1B. Mild acneiform lesions were also observed and considered incidental findings. This case highlights the importance of dermatologic assessment within a multidisciplinary diagnostic pathway, contributing to the recognition of subtle cutaneous phenotypes and the implementation of long-term photoprotection and surveillance strategies in patients with mild albinism.

## Introduction

Oculocutaneous albinism (OCA) comprises a group of inherited disorders characterized by reduced or absent melanin biosynthesis affecting the skin, hair, and eyes, with a broad clinical spectrum and variable phenotypic expression [1]. Among the OCA subtypes, OCA type 1 (OCA1) is caused by variants in the *TYR* gene, which encodes tyrosinase, a key enzyme required for melanin production [1]. The degree of residual tyrosinase function is clinically relevant, as it contributes to phenotypic variability across the OCA1 spectrum, including milder hypomorphic presentations in which cutaneous findings may be subtle and easily overlooked during routine dermatologic examination [2].

From a dermatologic perspective, reduced melanin content results in diminished natural photoprotection, predisposing individuals with OCA to cumulative ultraviolet-induced damage. This pathophysiologic basis supports the need for early implementation of strict photoprotection measures and structured long-term dermatologic surveillance, as reflected in the management plan described in this case. Although severe phenotypes may present with striking hypopigmentation, milder forms such as OCA1B can manifest with only minimal pigmentary changes, emphasizing the need for dermatologists to recognize subtle phenotypes and integrate clinical history with multidisciplinary findings [2,3]. OCA is considered a multisystem pigmentary condition in which ocular manifestations are often prominent and may drive the initial diagnostic suspicion; however, dermatologic evaluation remains essential for long-term surveillance and patient education [3].

Genetic testing has become central for confirming subtype, refining genotype-phenotype correlation, and supporting counseling in patients with suspected albinism. Cohort studies demonstrate that molecular confirmation can improve diagnostic precision and clarify subtype distribution, particularly in patients with mild or atypical presentations [4]. The molecular diagnostic series further highlights the diversity of pathogenic and likely pathogenic variants across OCA genes and underscores the practical role of genetic testing in routine clinical care [5]. Mechanistic genotype-phenotype studies additionally support that specific *TYR* variants may influence protein stability and enzymatic activity, providing a biologic basis for milder OCA1B phenotypes in which dermatologic manifestations may be limited despite underlying molecular disease [6].

This case illustrates how multidisciplinary collaboration among ophthalmology, genetics, and dermatology is essential for accurate diagnosis and comprehensive management, particularly in patients with subtle phenotypic expression in whom isolated specialty evaluation may delay recognition.

## Case presentation

A previously healthy 16-year-old girl, born to non-consanguineous parents with darker phototypes (Fitzpatrick IV) [7], with no known family history of albinism or hypopigmentation, presented for evaluation. Early psychomotor development was normal and age-appropriate. At eight years of age, she began using corrective lenses due to progressive decreased visual acuity affecting distance vision. Over time, she reported visual fatigue and headaches associated with prolonged visual effort, prompting referral from primary care to ophthalmology.

Ophthalmologic evaluation documented reduced best-corrected visual acuity (BCVA), with uncorrected visual acuity of 20/100, improving to 20/50-20/60 with correction. Fundoscopic examination revealed pale optic discs and increased visibility of choroidal vessels in the retinal periphery. Suspicion of albinism increased at 14 years of age after optical coherence tomography (OCT) demonstrated the absence of foveal depression consistent with foveal hypoplasia, prompting referral to clinical genetics.

Genetic evaluation was performed using a commercially available next-generation sequencing (NGS) panel targeting genes associated with oculocutaneous albinism. Molecular analysis identified three variants in the *TYR* gene, associated with oculocutaneous albinism type 1B (OCA1B) [3]. Parental segregation studies were not performed; however, based on the combination of a likely pathogenic variant and two hypomorphic alleles, the molecular findings are consistent with an OCA1B phenotype. A heterozygous likely pathogenic variant, c.140G>A (p.Gly47Asp), was identified together with two heterozygous hypomorphic alleles, c.575C>A (p.Ser192Tyr) and c.1205G>A (p.Arg402Gln). This combination of a likely pathogenic variant together with two hypomorphic alleles has been associated with residual tyrosinase activity, providing a molecular basis for the milder OCA1B phenotype observed in this patient. Variant classification was performed according to the American College of Medical Genetics and Genomics/Association for Molecular Pathology (ACMG/AMP) guidelines using the reference transcript NM_000372.4 [8]. The molecular findings are summarized in Table 1.

**Table 1:** Molecular findings identified in the TYR gene using a targeted oculocutaneous albinism panel Genetic analysis was performed using next-generation sequencing (NGS) with exome enrichment targeting genes associated with oculocutaneous albinism (OCA). Variants were classified according to the American College of Medical Genetics and Genomics/Association for Molecular Pathology (ACMG/AMP) guidelines using the reference transcript NM_000372.4 [8]. Abbreviations: OCA, oculocutaneous albinism; NGS, next-generation sequencing; ACMG/AMP, American College of Medical Genetics and Genomics/Association for Molecular Pathology; *TYR*, tyrosinase gene.

Gene	Transcript	Nucleotide change	Protein change	dbSNP	Zygosity	Classification
TYR	NM_000372.4	c.140G>A	p.Gly47Asp	rs61753180	Heterozygous	Likely pathogenic
TYR	NM_000372.4	c.575C>A	p.Ser192Tyr	rs1042602	Heterozygous	Hypomorphic allele
TYR	NM_000372.4	c.1205G>A	p.Arg402Gln	rs1126809	Heterozygous	Hypomorphic allele

**Figure 1:**
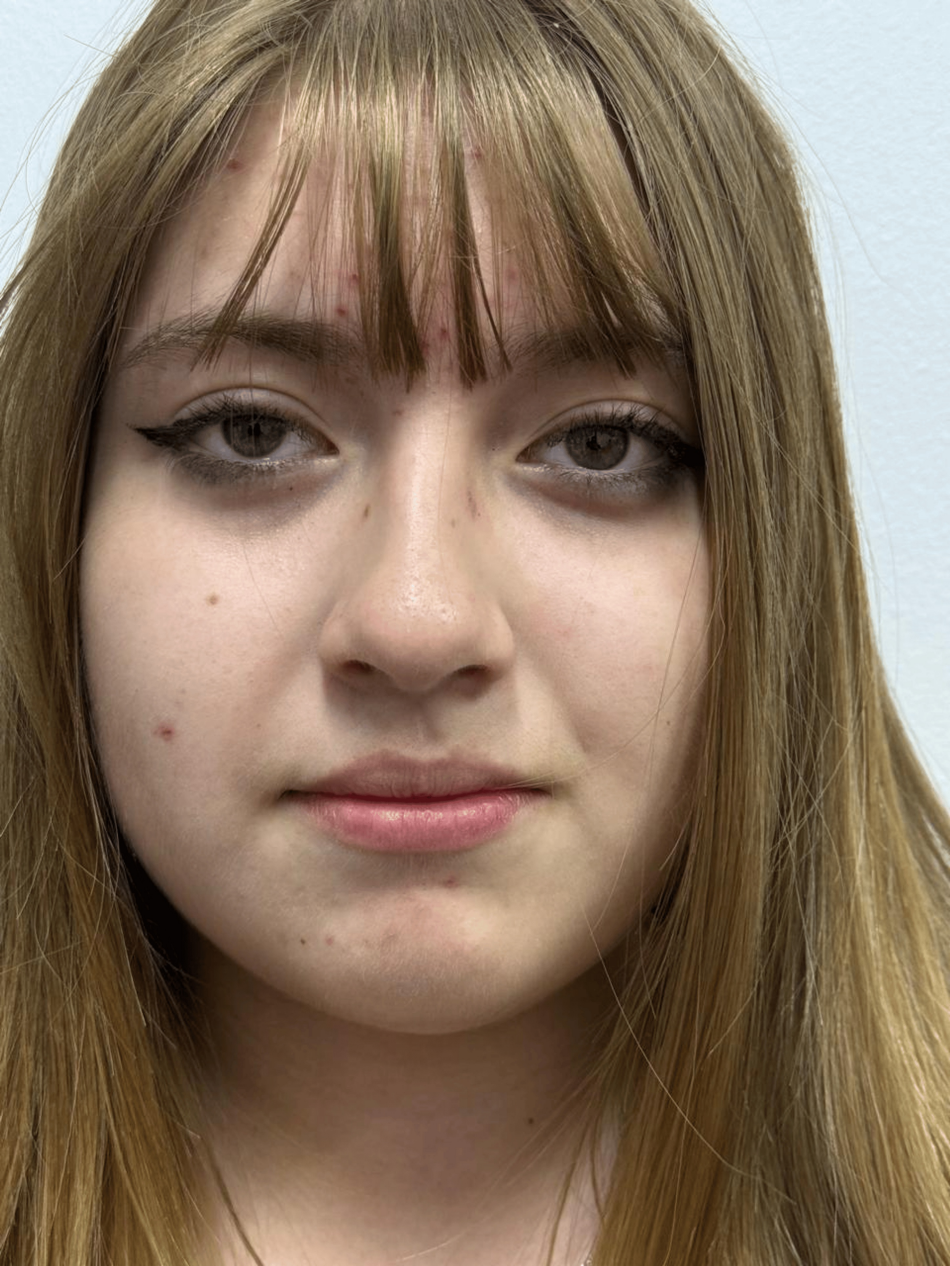
Facial phenotype and mild inflammatory acne in oculocutaneous albinism type 1B Facial clinical presentation demonstrating light hair pigmentation, gray-green irides, and mild inflammatory acne with excoriated papules predominantly on the forehead. All images were obtained with the patient's and guardian's consent for publication.

At 16 years of age, the patient was referred to dermatology following genetic confirmation. She denied cutaneous symptoms. Physical examination revealed Fitzpatrick phototype II with diffuse cutaneous hypopigmentation and gray-green irides with reduced pigmentation, consistent with a mild OCA1B phenotype. Additionally, mild facial acne, characterized by comedones and excoriated papules, predominantly on the forehead, was observed and considered an incidental finding not related to the underlying condition (Figure 1).

**Figure 2:**
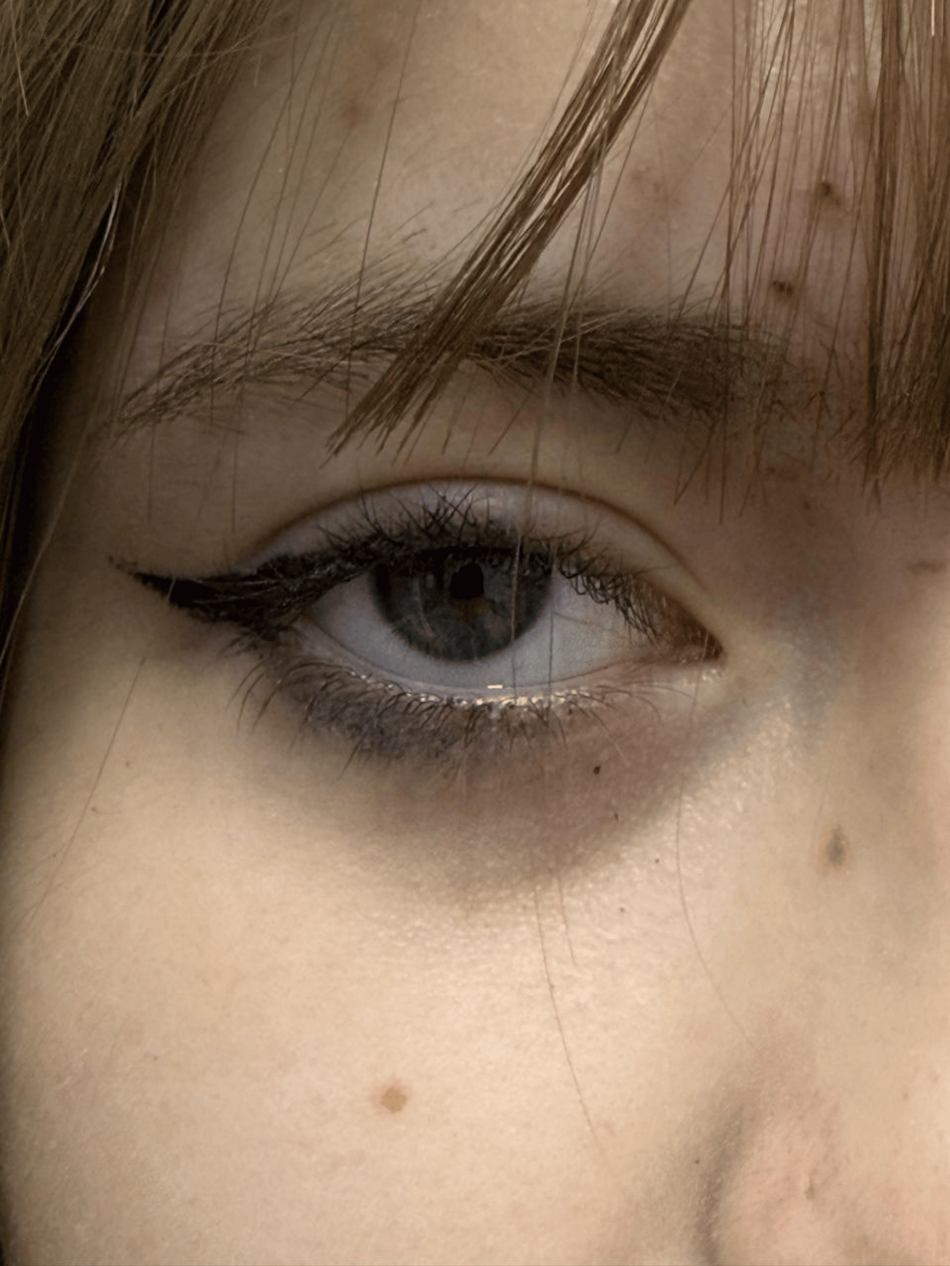
Gray-green iris pigmentation in a hypomorphic TYR-related phenotype Close-up image of the iris demonstrating gray-green pigmentation with reduced melanin density, compatible with a mild oculocutaneous albinism type 1B phenotype associated with *TYR* variants. All images were obtained with the patient's and guardian's consent for publication.

Close-up evaluation of the iris demonstrated gray-green pigmentation consistent with a mild hypopigmented phenotype associated with hypomorphic *TYR* variants (Figure 2).

**Figure 3:**
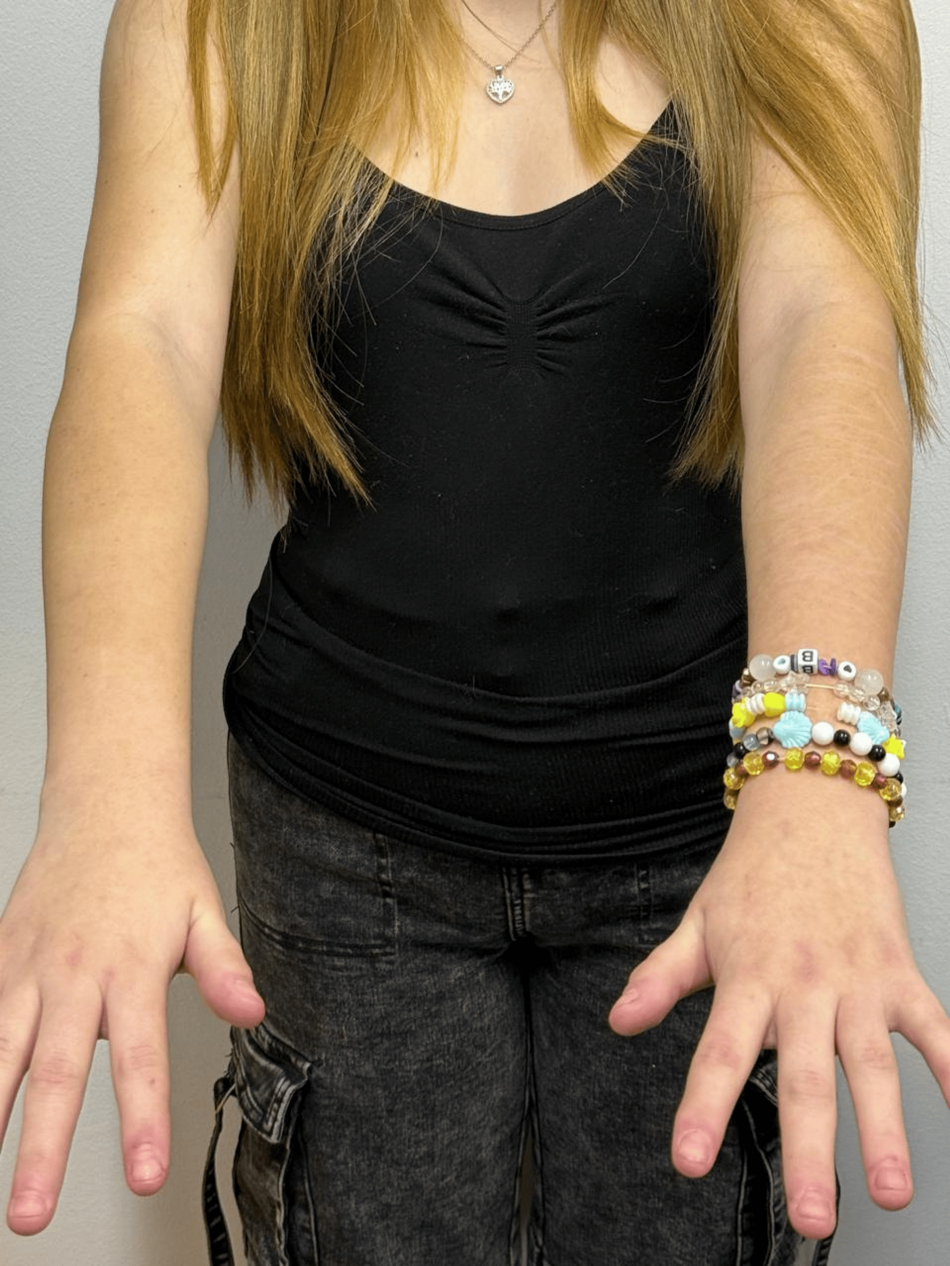
Generalized light cutaneous phenotype of the upper extremities consistent with Fitzpatrick phototype II Clinical image of the upper extremities showing a generalized light cutaneous phenotype consistent with Fitzpatrick phototype II without clinically suspicious melanocytic lesions or evident actinic damage. All images were obtained with the patient's and guardian's consent for publication.

Examination of the upper extremities showed a generalized light cutaneous phenotype consistent with Fitzpatrick phototype II, without clinically suspicious melanocytic lesions (Figure 3).

No clinically significant pigmentary abnormalities or suspicious lesions were observed.

Preventive counseling focused on strict photoprotection, and treatment with topical adapalene 0.1% was initiated. Standard acne management was applied, with particular emphasis on photoprotection given the patient’s increased susceptibility to ultraviolet-induced damage. Dermatologic follow-up every six months was recommended for nevus surveillance and monitoring of actinic damage.

## Discussion

Oculocutaneous albinism type 1 (OCA1) results from variants in *TYR*, which encodes tyrosinase, a key enzyme in melanin biosynthesis [1]. Experimental and functional evidence supports that impaired tyrosinase maturation and activity contribute to variable residual function and phenotypic heterogeneity across OCA1 [2]. Clinical reviews emphasize that oculocutaneous albinism is a multisystem pigmentary disorder in which ocular involvement is often prominent and may guide the initial diagnostic suspicion [3]. In clinical cohorts, molecular confirmation can refine subtype classification and support counseling, particularly when phenotypes are mild or atypical [4]. The molecular diagnostic series further demonstrates the diversity of pathogenic and likely pathogenic variants across OCA genes and the practical role of genetic testing in routine care [5]. Mechanistic genotype-phenotype work also links *TYR* variants to protein stability and enzymatic activity, providing biologic plausibility for milder OCA1B presentations when residual function is preserved [6].

Differences between OCA1A and OCA1B are summarized in Table 2.

**Table 2:** Clinical comparison between OCA1A and OCA1B Elaborated from clinical reviews and clinico-molecular studies of oculocutaneous albinism together with functional evidence on tyrosinase biology. Main sources include Oetting et al. [1], Halaban et al. [2], Grønskov et al. [3], Hutton and Spritz [4], Rooryck et al. [5], and Dolinska et al. [6].

Domain	OCA1A	OCA1B
Tyrosinase activity	Absent	Reduced/residual
Pigmentation over time	Minimal or absent	Partial increase possible
Hair/skin phenotype	Marked hypopigmentation	Variable; may be subtle
Iris color	Typically very light	Variable (may be hazel/green/gray)
Ocular features	Often pronounced	Variable; may be detected early with imaging
Dermatologic strategy	Strict photoprotection + surveillance	Strict photoprotection + surveillance

In our case, the presence of residual pigmentation, gray-green irides, and relatively mild cutaneous hypopigmentation is consistent with the OCA1B phenotype, in contrast to the near-complete absence of pigmentation typically observed in OCA1A.

From a clinical standpoint, additional manifestations in OCA can include photophobia, nystagmus, strabismus, refractive errors, and reduced visual acuity, reflecting abnormal foveal development and optic pathway changes [3]. In our case, the diagnostic pathway was ophthalmology-initiated, supported by fundoscopic findings (pale optic discs and visible choroidal vessels) and OCT evidence of absent foveal depression, followed by molecular confirmation and dermatologic preventive evaluation. The real-world dermatologic risk in albinism is driven by cumulative ultraviolet exposure and reduced melanin-mediated photoprotection. Histopathologic series in African individuals with albinism demonstrate a substantial burden of skin cancers, frequently involving sun-exposed sites and dominated by keratinocyte carcinomas [9]. Additional reports from sub-Saharan Africa emphasize the disproportionate risk of squamous cell carcinoma in contexts of intense ultraviolet radiation and limited access to photoprotective measures [10]. While these data may not be directly generalizable across all geographic settings, they underscore that risk is strongly modified by environment, behavior, and healthcare access. In this context, the patient’s mild phenotype and access to dermatologic care may influence her individual risk profile, supporting the need for personalized preventive strategies. Comprehensive dermatology reviews further synthesize genetic, clinical, and psychosocial aspects of albinism and reinforce the importance of continuous photoprotection and periodic surveillance to reduce long-term morbidity [11]. Observational work on photoprotective behavior among persons with albinism highlights gaps in awareness, access, and adherence, supporting a role for repeated counseling and structured prevention plans rather than one-time advice [12]. A broader literature review of skin cancers in people with albinism confirms that most reported malignancies are squamous cell carcinoma and basal cell carcinoma, with melanoma less frequent but still reported, reinforcing the need for ongoing surveillance rather than reliance on perceived “low melanoma risk” [13]. Finally, contemporary genetic studies continue to expand the molecular landscape of OCA and provide additional context for interpreting genotype-phenotype relationships in clinical practice [14].

In mild OCA1B phenotypes, dermatologic findings may be subtle, potentially leading to underestimation of cumulative ultraviolet risk. Clinicians should maintain a low threshold for monitoring early signs of actinic damage, including lentigines, actinic keratoses, and evolving melanocytic lesions, even in patients with subtle phenotypes. Early dermatologic involvement remains clinically valuable to implement strict photoprotection, establish longitudinal follow-up, and maintain a low threshold for evaluation of evolving lesions, particularly in adolescents who may accumulate ultraviolet exposure over decades. Taken together, this case highlights several practical clinical considerations relevant to dermatologic practice. Mild OCA1B may initially present with ophthalmologic findings rather than overt cutaneous signs, and structural imaging such as OCT can support early diagnostic suspicion. Integration of ophthalmologic evaluation, molecular confirmation through NGS, and dermatologic assessment allows timely recognition of hypopigmented phenotypes and facilitates the implementation of preventive strategies. Even when cutaneous manifestations are minimal, structured photoprotection and long-term dermatologic surveillance remain essential components of care aimed at reducing cumulative ultraviolet damage and potential skin cancer risk. From a genetic counseling perspective, recurrence risk within the family depends on parental carrier status. In autosomal recessive conditions such as OCA1B, when both parents are obligate carriers, as suggested in this case, the risk of recurrence for future offspring may reach approximately 25%, underscoring the importance of multidisciplinary collaboration between dermatology, ophthalmology, and clinical genetics in comprehensive patient and family management.

## Conclusions

This case illustrates a mild phenotype of OCA1B in which dermatologic findings were subtle despite a confirmed molecular diagnosis. Recognition of hypopigmented phenotypes with minimal cutaneous manifestations remains important in dermatologic practice, as reduced melanin content may still confer long-term susceptibility to ultraviolet-related damage. A multidisciplinary diagnostic pathway integrating ophthalmologic assessment, genetic confirmation, and dermatologic evaluation allowed early implementation of preventive strategies. Even in patients with limited clinical signs, structured photoprotection and longitudinal dermatologic surveillance represent key components of management aimed at reducing cumulative actinic injury and potential skin cancer risk.
